# Ethics—Dr. Hammond and the Record

**Published:** 1874-11

**Authors:** 


THE MEDICAL PROFESSION may, in some sort, be likened to a great family of brethren, linked together by the ties of a common consanguinity, having all a similar mission, pursuing all like ends, all alike interested in the maintenance of the dignity, the honor, the chivalric *esprit de corps* of an ancient and noble lineage. It should be the pride, and the pleasure also, of every son of the great household to vie with his fellows in his efforts to keep the family escutcheon without spot and blameless; to hold the family arms in highest esteem and reverence; to bear aloft the banner of his house in the battle of life, and plant it firmly upon higher, and yet higher ground. Mean and selfish indeed must be the heart which does not throb with something of enthusiasm at the touch of such influences when the bugle of his clan calls him to action. How ignoble the one who lingers behind to lay up to himself sordid pelf while duty loudly calls to the front. How criminal he who for selfish gain wou'd stab to the heart his comrades in the line of their duty.

An unprejudiced mind can scarcely contemplate the Code of Ethics of the American Medical Association and fail to be struck with the chivalric spirit of devotion to the honor and dignity of the profession, as well as the true and enduring welfare of its individual members, which everywhere pervades it. In common with all human laws, the medical code is certainly not without its imperfections. The artful and designing may, and often do, wrest it to the covering of their nefarious practices and selfish schemes; but however much the *letter* of the law may kill in the hands of the unworthy, all must acknowledge that its pure and beneficent *spirit* is full of joy and hope and life. In all civilized countries, and in all ages of the world, legislative wisdom has busied itself year by year, generation after generation, in perfecting and strengthening statutory law, and still the guilty go unpunished. There can be no perfection of human law until mankind shall have reached a point of moral elevation which shall make every man a law unto himself, for the rightful regulation of his intercourse with his fellows.

Viewed simply as a law for the regulation of professional conduct and intercourse, as an instrument for the punishment of transgressors, the code of ethics is sadly deficient; it falls far short of the design of its framers. Times without number has it been made the pretext for contention, wrangling and strife, for heart-burnings, for envy and malice, and for all uncharitableness.

Often is its letter perverted to the utter destruction of the harmony and usefulness of our medical bodies, and this is gross violation of its conciliatory and beneficient spirit. Upon the other hand when we view the code in the light of a written expression of the *animus* which the profession as a body would inculcate in, and enjoin upon, its individual members, it challenges both our respect and our admiration.

Earnest, and we think very just, complaint has often been made, that whilst the smaller men in our profession are held rigorously to the letter of the law, those who occupy positions of eminence and of influence are in the constant practice of gross violations of the spirit of the code. Nor does the complaint stop here; it is confidently asserted that certain parties, by reason of their high position and commanding influence, are enabled to set at naught the plain provisions of the written law with impunity.

Just now a fierce controversy is going on between Professor Hammond, of New York, and the editor of the *Medical Record*, growing out of some severe strictures upon the professional conduct of the former in the editorial columns of the *Record*. It is foreign to our present purpose to lead our readers into the discussion of the merits of this unfortunate wrangle, or to ask them to side with either the one or the other party. Suffice it to say that Prof. Hammond saw proper to publish in the columns of the New York *Daily Tribune*, a lengthy account, illustrated with wood cuts, of certain microscopical discoveries which he supposed himself to have made in the brain and cord of individuals who had died, as he believed, of hydrophobia. The editor of the *Record* took occasion to animadvert rather harshly upon Dr. Hammond's assumed facts, as well as the spirit in which they were presented, and the channel selected by him for bringing himself before the public. A demand was made by Dr. Hammond for redress; Dr. Shrady offered him the columns of the *Record* for his defence. Declining this offer, he appealed to the publishers, who referred him back to the editor, and a civil action in the courts for defamation of character was the result.

In striking contrast with that of Dr. Hammond is the spirit manifested by Sir James Paget in the remark: "I venture to hope that I am not thought likely to be guilty of encouraging the appearance of my name in newspapers."

It cannot be denied that the practice of *encouraging* the appearance of the names of otherwise reputable physicians in the newspapers is by far too common at the present time. Some give this encouragement simply by "cultivating" the editors, and especially the reporters of newspapers; others by lounging about the street corners and "blowing" the passers-by; others frequent the editorial sanctum, and improve the opportunity to "gas" of their professional prowess; others rush in with unseemly haste, and offer for inspection a lengthy visiting list by way of apology for the absence of ceremony; others lament that they are daily besieged by invalids from distant counties and States, who give them no rest. There are those, very conscientious ones, who feel in duty bound to go further, and lay before the interested public their peculiar views upon any prevailing epidemic, and their special methods of treatment, which are always so successful, you know. Few, very few, of any repute as yet, we are glad to say, venture so far, as Dr. Hammond has done, as to enter into scientific disquisition and furnish wood cuts for illustration, that the newsboys may be enabled to imbibe the mysteries of nerve-pathology.

The newspaper is the chosen vehicle through which nostrum vendors, quacks and charlatans are daily accustomed to trumpet to the gaping populace their marvelous cures and wonderful achievements. The medical profession is not as yet prepared to step down from its dignified and elevated position to seize the weapons of the quack and "fight the devil with fire."

Whilst the profession cannot give countenance, in any sense, to the disreputable practices to which we allude, and whilst it is the duty of the medical press to note and condemn such infractions of the code, it does not follow that the ill-tempered and unmeasured denunciations of medical editors, (to say nothing of occasional indulgence in billingsgate,) are either to be applauded or approved. The latter, not unfrequently, do as gross violence to to the spirit of the code as do those who infringe upon its letter.

No state of circumstances, we apprehend, can justify a resort to the courts for the adjudication of a professional quarrel, such as that under consideration. We rejoice that we live in a region where a better remedy exists for private and delicate grievances than the scandal of the courts. The profession of the South can have no sympathy for Dr. Hammond in the alternative he has chosen.

We find in the *American Medical Weekly* the following just tribute to our valued friend and former confrere:

Dr. J. P. Logan, of Atlanta, has retired from his chair of Clinical Medicine in the Atlanta Medical College. His loss from the didactic collegiate field will be as severely felt as was his retirement from the field of medical journalism. Like all men of merit, he blushes at the truth spoken of him, but even if there be a genuine facial cremation, he must read this: "*non tettigit non ornavit.*"

